# Mouse Intratongue Injection With Cancer Cells—An Orthotopic Model for Head and Neck Squamous Cell Carcinoma

**DOI:** 10.21769/BioProtoc.5861

**Published:** 2026-11-05

**Authors:** Gautier Follain, Jasmin Kaivola, Omkar Joshi, Mathilde Mathieu, Johanna Ivaska

**Affiliations:** 1 Turku Bioscience Centre, University of Turku and Åbo Akademi University, Turku, Finland; 2 Faculty of Science and Engineering, Cell Biology, Åbo Akademi University, Turku, Finland; 3 Turku Collegium for Science, Medicine and Technology (TCSMT), Turku, Finland; 4 InFLAMES Research Flagship Center, University of Turku and Åbo Akademi University, Turku, Finland; 5 Foundation for the Finnish Cancer Institute, Tukholmankatu 8, FI-00014 Helsinki, Finland

**Keywords:** Preclinical cancer model, Orthotopic mouse model, Head and neck squamous cell carcinoma (HNSCC), Intratongue injection, Tumor microenvironment, Tumor growth, Tumor invasion

## Abstract

Syngeneic and orthotopic injection of cancer cells in mice is a gold standard model in pre-clinical cancer research. Herein, we detail a protocol of orthotopic intratongue injection as a model for upper respiratory tract cancer studies. This model recapitulates tumor growth and invasion in an orthotopic site, the oral cavity mucosa. It can be experimentally designed in a syngeneic context or used to study patient-derived cells in immunocompromised animals. Procedurally, mice are anesthetized and injected with a small volume of cancer cell suspension near the lateral border of the tongue, right under the mucous membrane. Post-injection, animal welfare is carefully attended to by appropriate analgesia, while providing soft food for the duration of the experiment to ensure the ability of the mice to eat ad libitum. Tongues and potential metastatic organs are dissected and can be sampled and analyzed using relevant state-of-the-art methods, including -omics and imaging approaches. We describe a broad range of distinct tumor profiling and analysis methods applicable to this model. These include physical measurements of elasticity with atomic force microscopy on fresh tissue, hematoxylin-eosin staining and standard immunohistochemistry, and confocal microscopy applied to paraffin-embedded tongue sections. This model is orthotopic, accessible without surgery, easy to monitor, and rapid to perform.

Key features

• Orthotopic and clinically relevant head and neck squamous cell carcinoma (HNSCC) model.

• Rapid, reproducible, and minimally invasive procedure.

• Compatible with multimodal downstream analyses.

• Versatile platform for preclinical studies.

## Background

This protocol was inspired by previous work [1–3] demonstrating the preclinical relevance of tongue orthotopic injection in the study of head and neck cancers. Myers et al. [1] implanted human oral cancer cell lines directly into the tongue, where tumors formed more efficiently and more closely reproduced the histology and metastatic behavior of human disease than more classic subcutaneous models. Of note, this observation was confirmed and further studied recently from an immune landscape perspective [4]. Myers’ seminal work accelerated the development of metastatic progression studies, either focusing on the local tumor microenvironment characterization [5–7] or the factors influencing the formation of lung metastases. As such, Benaich et al. [2] focused on the role of miR-203, and Pascual et al. [3] studied how lipid metabolism affects survival and metastatic initiation.

Our protocol was developed and optimized for the completion of two studies: Punovuori et al. [8] focused on the contribution of extracellular matrix composition and cancer-associated fibroblasts in tumor progression, and Kaivola et al. [9] focused on the role of tissue stiffening in vocal fold cancer progression. We employed mouse intratongue injection as an in vivo model system to compare the growth of advanced-stage T3 head and neck squamous cell carcinoma (HNSCC) cells and their therapeutic response to treatment with a clinically relevant YAP-TEAD inhibitor. We also recently used this protocol in Punovuori et al. [8], where we reprogrammed cancer cells through cancer-stroma (HNSCC cell–cancer-associated fibroblast) crosstalk in vitro using spheroid co-cultures and compared the growth of two distinct HNSCC models injected in mice tongues with or without cancer-associated fibroblasts.

Most previously reported applications of intratongue (also called intralingual) injection involve injecting cell-free solutions of chemicals (like sodium selenite [10] or choleratoxin B-saporin [11]), prions [12], or virus particles (AAV), often in the context of optogenetic studies [13–18]. Further, it has also been used for acute pain studies using large needles and volumes under isoflurane anesthesia [19,20].

In contrast, there are only a few carcinogenesis [21] and tumor xenograft studies published for this orthotopic injection site, first proposed by the Fidler lab [1] (2002). Technically, downstream applications involved histology (tissue staining, immunohistochemistry, and immunostaining) and whole-animal non-invasive imaging (bioluminescence- or fluorescence-based). Intravital imaging [22] was also applied to the tongue using a non-invasive holder and 2-photon illumination post-intratongue injection to study tumor invasion and intravasation [23,24]. Of note, the experimental endpoint post-injection varies from two weeks to several months, depending on cell lines and animal models, as well as the variable cell numbers used. Lastly, similar to the orthotopic injection protocol described here, intramucosal injection in the mouse cheek has been described [25]. However, to the best of our knowledge, a detailed protocol for intratongue injection is still lacking. We believe that our refined protocol is compatible with high reproducibility and aligns extremely well with current ethics on animal welfare worldwide.

As a brief description of the protocol (Figure 1A), mice are first treated with analgesic and anti-inflammatory drugs. The injection mix for the intratongue injection is prepared. The mice are anesthetized with a ketamine/xylazine mix that will keep them unconscious for approximately 40 min. When ready, the injection needle is prepared, and the mice are suitably positioned to enable smooth intratongue injections (illustrated in Figure 1C). The injection procedure itself takes less than 1 min per animal. Mice are monitored until the anesthesia subsides. Analgesic and anti-inflammatory treatment is administered 6 h post-intratongue injections and continued for the following three days. Fresh, softened food is provided daily throughout the experiment. Cancer progression is closely monitored until the experimental objective is achieved and/or the animal reaches the humane endpoint. As this protocol includes injection inside the mouth, we typically observe a modest, transient decrease in body weight shortly after injection. Mice usually recover the lost weight in the first week post-injection. Then, when approaching the endpoint, animals lose weight rapidly and irreversibly (Figure 1B). After sacrifice, the tongues and potential metastatic organs are harvested and processed. As a control, in addition to the experimental controls, two non-injected animals are included to provide a reference to normal tissue.

**Figure 1.**
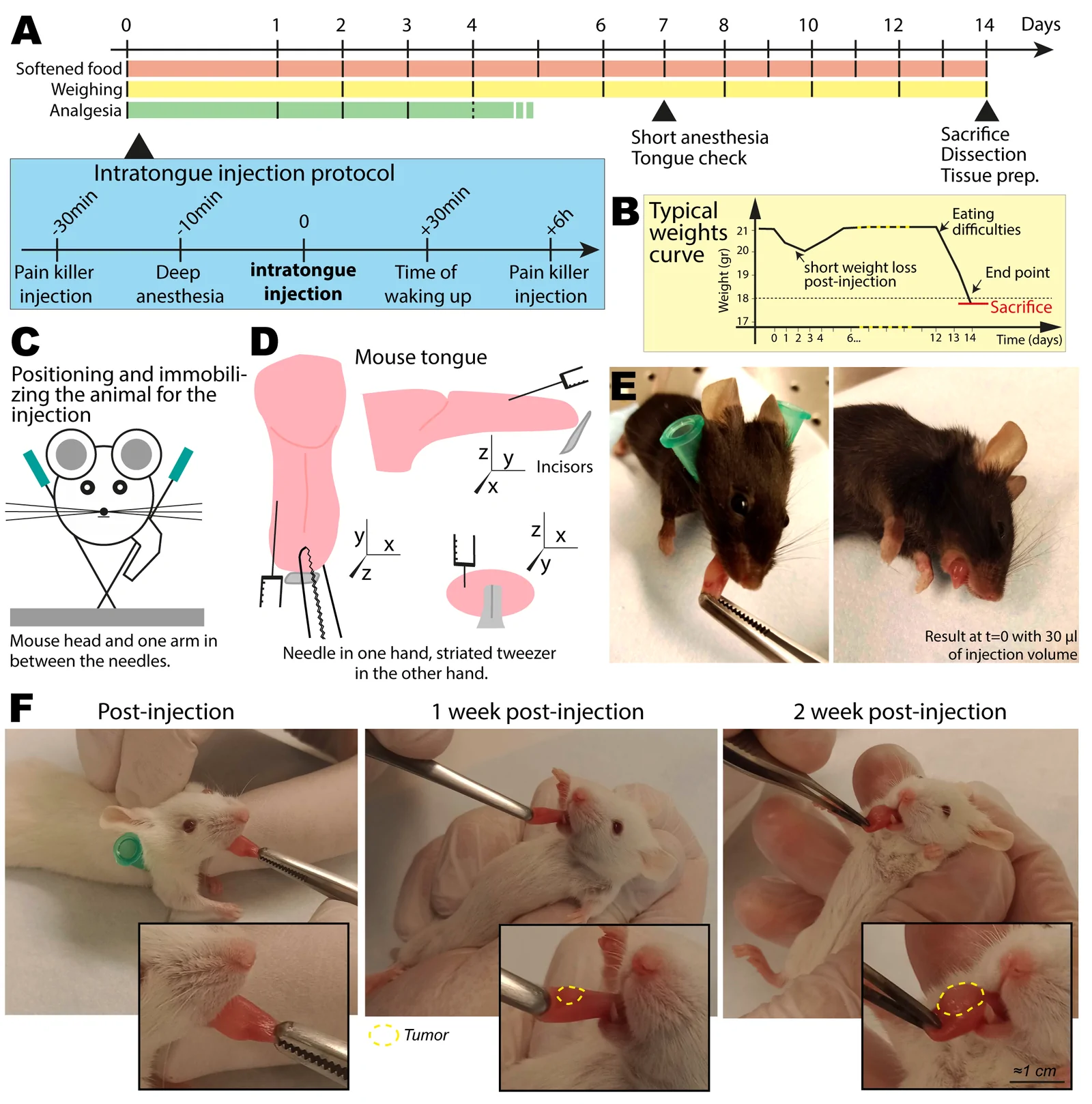
Experimental design for intratongue injection and subsequent monitoring. (A) Experimental timeline and summary of key steps. (B) Typical weight curve of the animals during the protocol. (C) Schematic of the position of the animal on the holder for intratongue injection. (D) Schematic of the tongue injection spot from three angles. The injection needle comes nearly parallel to the tongue, on the side, and the needle does not enter more than 2 mm into the tissue to stay in the submucosal compartment. (E) Representative pictures of the mouse tongue, directly post-injection (C57BL/6 mouse). (F) Representative pictures of the mouse tongue during the monitoring of the tongue (NSG mouse): directly post-injection, after 1 week, and after 2 weeks (endpoint).

Based on our expertise and existing literature, intratongue injection in mice is a relevant experimental system in research contexts ranging from fundamental to pre-clinical research. The tools, methods, and technical tips presented here are applicable to any tongue injection, with or without cells. In cancer research, it should be used as an orthotopic site of injection for upper respiratory tract cancer models. As previously reported [26], growing HNSCC lines in classical subcutaneous assays in the flank of mice is challenging. In our hands, all tested HNSCC cell lines grew a tumor in the tongue [8,9]. However, it has, a priori, no added value for other cancers, in which other orthotopic sites or more classical subcutaneous injection are more relevant and/or safer for the animals.

Our protocol is compatible with any kind of cellular mixes (cancer, immune, or stromal cells, separately or together). We believe that the addition of Matrigel is instrumental for the early engraftment of the tumor cells post-injection, but one can work without it if the cellular model allows. If well-positioned, liquid solutions do not leak out of the tongue when you pull the needle out. This model is suitable for distinct systemic pre-treatment or treatment during the experiment, and therapeutic agents can be administered in different ways (drinking water, gavage, injections, etc.). Although we did not perform live imaging, this approach is possible after the injection, as mentioned earlier (using bioluminescence, intravital imaging, etc.). Furthermore, tumor growth can be observed visually and with caliper measurements of animals under low isoflurane anesthesia. Visual inspection enables excellent quality control and evaluation of the progression of the experiment. After injection, the administered Matrigel–cell mixture appears like a small translucent lentil. After a week, a small whiter spot appears on the tongue, which grows over the weeks. Post-sacrifice, tongue dissection is relatively simple if well executed, and sample processing complexity is dependent on the experimental goal. Lastly, although depending on the aggressiveness of the cancer cell line and the cell number injected, the experiment duration will vary [9]; animals in our study reached the clinical endpoint in two weeks (precisely reproduced in independent experiments). We believe this is the shortest time to be expected.

This protocol requires training in animal experimentation. It uses intraperitoneal, subcutaneous, and intratongue injections. Experimenters must be familiar with animal anesthesia procedures and safety. It also requires a good appreciation of animal signs of discomfort, and significant dedicated time for animal monitoring (regular weighing and daily softened food change). Toward the endpoint of the experiment, mouse health will deteriorate very quickly; thus, careful and thorough monitoring is necessary. We recommend following the OBSERVE consensus guidelines to monitor the animals [27]. Based on our experience, this protocol is non-trivial and should be performed by advanced animal experimenters with potential support from animal facility personnel. Of note, the injection protocol presented here does not strictly require the presence of a second experimenter, although it is generally advisable to work in pairs during animal experimentation, as it can help during certain steps.

This protocol is not compatible with resection of the primary tumor for long-term relapse studies. Tumor growth impairs the animal’s capacity to eat and, ultimately, to breathe. Animal pain signs are visible soon after the injection (prompting treatment with analgesics) and are later replaced by low-level animal stress (hyperactive animals, sometimes more aggressive than non-injected counterparts). These are ethical, as well as experimental, concerns that must be considered before starting the experiment, with a clear definition of endpoints.

Last, since this procedure includes animal experimentation, all experiments should be performed in accordance with relevant guidelines and regulations. Our protocol was ethically assessed and authorized by the National Animal Experiment Board and in accordance with the Finnish Act on Animal Experimentation (animal license numbers ESAVI/6253/2024).

In this article, we present standard histology, classical fluorescence imaging modalities (widefield and confocal), and atomic force microscopy (AFM) measurements as examples. Indeed, this protocol does not detail the processing of the sample after dissection, including paraffin embedding, sectioning, and staining, as these are standard and are out of the scope of this article. We used classic histology approaches that are standard for many organs, standard immunofluorescence staining approaches, and published AFM on the fresh tissue slice protocol [28]. Finally, it also does not detail standard drug administration procedures via intraperitoneal injection, subcutaneous injection, or oral gavage.

## Materials and reagents

**Biological materials**

1. UT-SCC-103 cells (T3 human glottic laryngeal cancer, Turku University Hospital) were used in [9]. The cell line was generated at the Turku University Hospital and has undergone scientific evaluation by Auria Biobank with a positive decision of release (AB22-7195) to be used in the study.

2. Mouse strain lines tested include NSG mice (NOD.CB17-*Prkdcscid*/NCrHsd), Athymic nude mice (Hsd:Athymic Nude-*Foxn1nu*), and Black/6 mice (C57BL/6NHsd) from Inotiv (Netherlands). Immuno-competence and species compatibility must be taken into account to ensure the success of the experiment. NSG mice and human cells were used in [9].

**Reagents**

1. Ketamine, Ketaminol^®^ vet 50 mg/mL, 20 mL (MSD Animal Health, VNR: 511485)

2. Xylazine, Rompun^®^ vet 20 mg/mL, 25 mL (BAYER, VNR: 148999)

3. Buprenorphine, Bupaq^®^ multidose vet 0.3 mg/mL, 10 mL (Richter pharma, VNR: 563931)

4. Carprofen, Carprofelican^®^ vet 50 mg/mL, 20 mL (Dechra, VNR: 387854); store at 4 °C

5. Isoflurane, Attane vet 1,000 mg/g, 250 mL (VETMEDIC, VNR: 170579)

6. Sterile Dulbecco’s phosphate-buffered saline (PBS), 500 mL (Biowest, catalog number: L0615-500)

7. Sterile water; autoclaved tap water (121 °C, 20–30 min)

8. Ethanol, 80% (A12T 80, 5 L, Berner)

9. Formalin solution 10% phosphate buffered pH 7, ready filling solution (formaldehyde 4%) (Oy FF-Chemicals Ab, catalog number: FF119); store at 4 °C.

**Caution:** Formalin is toxic. Follow safety guidelines provided by the manufacturer.

10. Softened food: Normal food used for breeding [in our study, Teklad global 16% protein, 2916C (inotiv), is mixed in a 1:1 ratio with sterile water and left overnight at 4 °C to soak]

**Critical:** Mice might have difficulty to eat just after the injection; softened food is an easier intake form.

11. Cell culture media from the cell line of interest; in our study, we used basal media: DMEM (1×) + GlutaMAX^TM^-I, 500 mL (Gibco, catalog number: 31966-021), 10% fetal bovine serum (Biowest, catalog number: S1810-500), 1% penicillin-streptomycin (Gibco, catalog number: 15140-122), 1% non-essential amino acid (MEM NEAA) (Gibco, catalog number: 1140-035)

12. Trypsin-EDTA solution 10× (Sigma-Aldrich, catalog number: T4174)

13. Matrigel^®^ growth factor reduced (GFR) basement membrane matrix, LDEV-free, 10 mL (Corning, catalog number: CLS354230); store at -20 °C as 100-μL aliquots; keep on ice for thawing.

**Critical:** Do not freeze/thaw.

14. Optimal cutting temperature compound (Tissue-Tek, catalog number: 62550-01)

15. Poly-L-lysine solution (Sigma-Aldrich, catalog number: P4707)

16. cOmplete EDTA-free protease inhibitor cocktail (Sigma-Aldrich, catalog number: COEDTAF-RO)

17. Eye ointment to prevent desiccation during long anesthesia (Collyre, Bepanthen PRO)

**Laboratory supplies**

1. Classic small labware to prepare solutions (pipettes, tips, propipettes, stripette, glass container, 50 mL tubes, 15 mL tubes, Eppendorf tubes, etc.).

2. Cell culture material (pipettes, culture dishes, cell counter, centrifuge, …)

3. Insulin syringe for intratongue injection: 30G × ½” (0.3 mL, 0.30 mm) × 8 mm (any)

4. 1 mL syringe (Henke-ject Luer, HENKE SASS WOLF, 5010-200V0)

5. 30G needle for intraperitoneal (IP) and subcutaneous (SC) injections: Microlance 3, 30G × ½” (0.3 × 13mm) (BD, catalog number: 30400)

6. 21G needle for intratongue injection, as holders (Figure 1C): Henke-ject, 21G × ½” Nr. 2 (0.8 × 40 mm) (TW/LB, HENKE SASS WOLF, catalog number: 4710008040)

7. Harvested organ containers: 30 mL universal container, no label, screw-cap, sterile (DWK Life Sciences Limited, catalog number: 327152)

## Equipment

1. 200-mL beaker to make softened food (any)

2. Small bowl for softened food, 1/animal cage (any)

3. Spoon/spatula to mix and put softened food in cages (any)

4. Striated and large forceps (to hold the tongue) (standard pattern forceps) (Fine Science Tools, catalog number: 11000-14)

**Critical:** Preferably, choose an old one with unsharp striation and a 3-mm width minimum. Sharp striation can easily damage the tongue if you pinch too hard. Flat forceps will easily slip, so you need a stronger grip, which will also damage the tongue.

5. Large forceps (standard pattern forceps) (Fine Science Tools, catalog number: 11000-12)

6. Fine forceps, Dumont #5 fine forceps (Fine Science Tools, catalog number: 11254-20)

7. Large scissors, straight/sharp/blunt (Fine Science Tools, catalog number: 14001-13)

8. Fine scissors, straight, 11.5 cm (Fine Science Tools, catalog number: 14060-11)

9. Isoflurane-based anesthesia chamber (any)

10. Warming animal mattress (any)

11. Small animal scale (any)

12. CO_2_ chamber for sacrifice (any)

13. Stereomicroscope (Zeiss, model: Axio Zoom.V16)

14. Marianas spinning-disk imaging system with a Yokogawa CSU-W1 scanning unit on an inverted Zeiss Axio Observer Z1 microscope, with an OrcaFlash 4 sCMOS camera (Hamamatsu Photonics)

15. Slide scanner (Panoramic 1000 Digital Scanner, 20× NA 0.8 objective, 3DHISTECH).

16. Atomic force microscopy system, JPK NanoWizard II system (Bruker Nano GmbH) with a CellHesion module mounted on a Zeiss LSM510 confocal microscope (Carl Zeiss NTS Ltd.) with silicon nitride cantilevers (spring constant: 0.06 N/m, spherical 4.5 μm diameter tip) (Novascan Technologies)

17. Phone camera or other handy instrument to take pictures during monitoring (any)

## Procedure

**A. Before the experiment**

1. Plan the number of mice needed in total and per experimental group. A minimum of two extra non-injected mice should be included for normal tissue comparison (non-injected, sacrificed at the end of the experiments; see **General note 1**). Identify your animals in the cages. Accordingly, get enough drug stock and calculate your dilutions (see section B) and prepare the right number of needles, syringes, and routine labware. Minimize the number of last-minute tasks as much as possible to optimize the intratongue injection day.

2. Prepare softened food the day prior to the injection. Put hard pellets of normal animal food in a beaker and cover it with sterile water (1:1 approximately). Leave it overnight at 4 °C to achieve a porridge-like texture (softened food).

**Critical:** The next day, mix well and add two soup spoons into a small container to each cage of five mice. This is enough, as fresh, softened food will be provided every day. The exact quantity to prepare depends on the number of animals in the experiment. We recommend counting 4 pellets/cage of 5 animals and adjusting if needed.

**B. Intratongue injection day**

1. Prepare the animal experimentation room: Ensure a good spot for the intratongue injection with a warm mattress on one side and space to prepare the injection needles on the other side. In the center, prepare the mouse holder: stick two 21G needles in an X-shape in a polystyrene plaque (Figure 1C). This spot must be comfortable and spacious to inject the animals in the best condition. Place the striated large forceps (preferably an old pair) and the 30G syringes on the bench. In another corner of the room, keep the mouse cages and the materials to inject the painkiller and anti-inflammatory mix (PAI mix), the anesthesia mix (KX mix), and the drug stocks.

2. Prepare enough drug injection mixes for one experimental/intratongue injection group at a time:

a. Painkiller and anti-inflammatory mix (PAI): Buprenorphine 0.07 mg/kg and Carprofen 5 mg/kg in 100 µL of sterile PBS per mouse.

**Example for a group of 10 mice:**

i. Buprenorphine stock solution: 0.3 mg/mL = 0.03 mg/100 µL.

Aiming at 0.07 mg/kg of mouse = 0.0014 mg/20 mg, for our mice.

Stock dilution factor for 0.0014 mg/100 µL (injection volume): 0.03/0.0014 = 21

Prepare extra volume to a total of 1,200 µL: 1,200/21 = 57 µL of stock.

ii. Carprofen stock solution: 50 mg/mL = 5 mg/100 µL.

Aiming at 5 mg/kg of mouse = 0.1 mg/20 mg, for our mice.

Stock dilution factor for 0.1 mg/100 µL (injection volume): 5/0.1 = 500

Prepare extra volume to a total of 1,200 µL: 1,200/500 = 2.4 µL of stock.

iii. Solution preparation: 57 µL of Buprenorphine + 2.4 µL of Carprofen in 1,141 µL of sterile PBS.

b. Anesthesia mix (KX): Ketamine 75 mg/kg and Xylazine 6 mg/kg in 200 µL of sterile PBS per mouse (prepared as in step B2a).

c. Both diluted mixes can be kept on the bench at room temperature and used in the next couple of hours. Prepare fresh painkiller mix for each time point.

3. Perform intraperitoneal injection of the mice (one experimental group) with PAI mix (30G needles on 1 mL syringes) and leave the animals to rest in their cages for a minimum of 30 min before intratongue injection.

4. Prepare the cell/mix of cell suspension for injection in the tongue. The details of this step depend on the goal of the experiment. In our case, a single cell suspension of tumor cells was prepared. We adjusted the cell concentration to accommodate the later addition of Matrigel (step B7) to ultimately achieve 30,000 cells in 30 μL of media with 30% Matrigel (injection volume per mouse) (see **General note 2**). Keep the cell suspension on ice.

5. Subcutaneously inject the mice (one complete experimental group) with KX mix (30G needles on 1 mL syringes) and let the animals fall unconscious in their cages before transferring them to the warm mattress. This step should not take more than 5 min. Use the toe-pinch reflex test to verify that the animals are deeply anesthetized. See **Troubleshooting**.

6. Apply veterinary-grade ophthalmic ointment (e.g., Bepanthen Collyre) to both eyes of the animal to prevent eye desiccation due to long anesthesia.

7. Mix the Matrigel solution (kept on ice) with the cell suspension to achieve 30% Matrigel dilution in a final cell suspension volume of 30 μL. See **Troubleshooting**. (The use of Matrigel is optional; see **General note 3.**)

8. Place the first mouse on the (X-shaped) mouse holder. The head and one foreleg should be on top of the crossing needles to prevent the risk of choking (Figure 1C). For right-handed experimenters, the right foreleg of the mouse should be put with the head, and vice versa for left-handed experimenters.

9. Fill a 30G syringe with 30 μL of cell suspension to inject the tongue of the first animal.

10. Use your preferred hand to open the mouth of the mouse. Gently catch and hold the tongue of the mouse out of the mouth with the striated large forceps in your other hand.

11. Take the syringe in your preferred hand and inject at the spot shown in Figure 1D, near the lateral border. **Critical:** Insert the needle parallel to the tongue, 2 mm maximum into the tissue. The injection will create a small lump under the tongue mucosa. It will appear slightly translucent when compared to the rest of the tissue (Figure 1E). Pull out the needle and place the tongue back in the mouth of the animal. See **Troubleshooting.**

12. Place the animal back on the warm mattress and repeat steps B7–10 for all animals. These steps combined should not take more than 1 min per animal on the holder. Training for these steps on sacrificed animals is recommended.

13. After approximately 40 min, mice should wake up. Transfer them back to the cage. See **Troubleshooting**.

14. Six hours after the first injection of PAI mix, intraperitoneally inject the mice with PAI mix again (second dose of the day). Optimally, administer the second dose on the opposite side of the first (step B2).

15. Prepare softened food for the next day.

**C. Monitoring after intratongue injection**

1. For the next two days post-injection, there are three tasks (Figure 1A):

a. Check the animal’s health carefully (see OBSERVE guidelines, [27]). Because intratongue injection hampers the capacity to eat/drink, early death can happen within the first two days. See **Troubleshooting.**

b. Administer PAI mix twice a day (ideally more than 6 h apart).

c. Replace the softened food every day (and always prepare some for the next day). See **Troubleshooting.**

2. Starting from day 2 until the end of the experiment, weigh the animals every two days.

**Critical:** It is a good routine to check their health at the same time. See **Troubleshooting.**

3. Starting from day 2 until the end of the experiment, change softened food every day. This guarantees the freshness and softness of the food.

4. One week post-injection, perform the first tongue monitoring. Individually anesthetize mice with 2% isoflurane for 1 min (if possible, in a chamber; 1 L/min flow rate) for rapid anesthesia (effect lasts for approximately 1 min). Use your fingers to open the mouse’s mouth, catch the tip of the tongue, and pull it out with striated forceps. Observe/photograph the tumor site. See **Troubleshooting.** There should be a whitish area around the injected spot in the more pinkish tongue tissue (Figure 1F). It will be a smaller lump than at the time of injection and will not appear translucent anymore. This indicates successful engraftment. It is possible that, at this stage, there is no discernible white area visible on the tongue; however, the tongue can still contain a successfully engrafted tumor. This may be because the injection was performed slightly too deeply in the tissue.

5. Starting from one week post-injection, repeat the tongue monitoring every week until the endpoint (Figure 1A).

**Critical**: Direct visualization of the tumor is the best indicator to follow its growth.

**D. End of the experiment, sacrifice, and dissection**

1. The endpoint has to be defined in the animal license, following local recommendations. A large tumor volume and associated rapid and irreversible weight loss are the main signs. In [4], week 2 was the endpoint, but this may vary based on the cell line model and number of cells administered. See **Troubleshooting.**

2. Prepare all dissection tools, sterile PBS, and the harvested organ container, depending on the experimental objectives. In our case, some tongues were processed for histology and others for AFM (fresh-frozen samples). Consequently, we prepared containers with formalin (volume of fixative should be 20× the volume of the tissue), kept on ice, and with OCT for fresh snap-freezing, for histology and AFM, respectively.

3. Take mice individually to the CO_2_ chamber for sacrifice. The exact protocol of this step depends on the animal experimentation license. Of note, cervical dislocation might not be optimal as it will damage tissues close to the dissection.

4. Dissection steps are described in Figure 2. Briefly, cut through the neck skin to have access to the jaw muscles and bones (Figure 2A). Separate the bottom jaw from the top and flip it open on one side (Figure 2B) (see **Troubleshooting**). The tongue will be on the open surface of the bottom jaw (Figure 2C). Cut the tongue out and take some extra surrounding tissue (Figure 2D). Submerge it in cold sterile PBS (using a 10 cm petri dish as a container). Once out of the animal, clean the tongue sample of any blood spillage and refine the sample by removing potential rear teeth or other connective tissue stuck to it (see **Troubleshooting**). The tongue can be weighed at this stage as a tumor growth metric (Figure 3A). This takes less than 5 min per animal after a short training period. We recommend starting with the non-injected animals to retain a fresh memory of normal tissue. In case of very invasive cancer progression, this will enable better recognition of the non-normal tissue in the mouth and the possibility to adjust the dissection to harvest all cancerous material.

**Figure 2.**
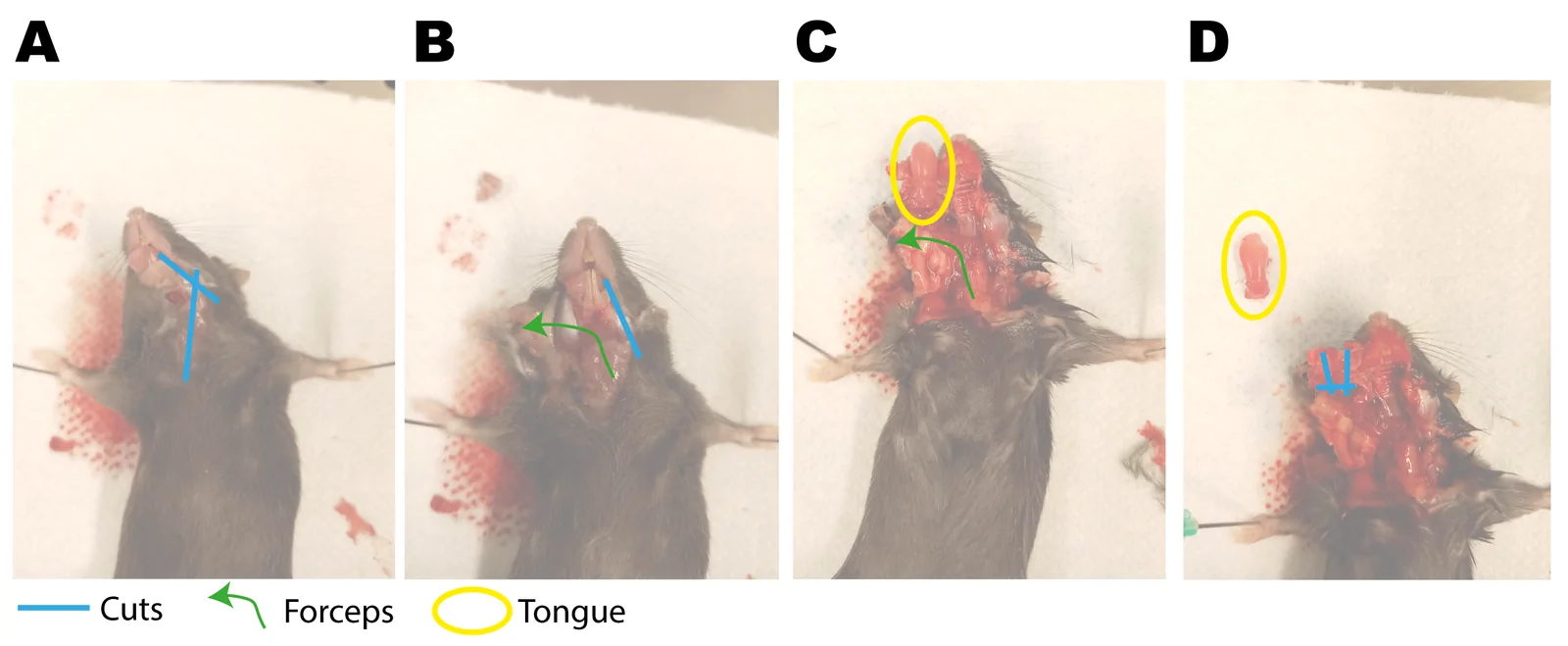
Dissection of the tongue. (A) C57BL/6 mouse. First, cut through the skin (blue lines) to reach the jaw muscles and bones. (B) Cut to detach the jaws. (C) Using tweezers, open the mouth (green arrow) to get access to the tongue (yellow oval). (D) Cut the tongue from the rest of the animal.

5. Drop the cleaned and refined tongue sample in formalin, kept on ice, or snap-freeze it in OCT. Repeat for each mouse.

6. Incubate the tongue in formalin for 24 h. Transfer to 70% ethanol and keep at 4 °C. For snap-frozen samples, store at -150 °C until use.

7. For formalin-fixed samples, we recommend taking a picture of the tongues at this stage with a stereomicroscope (Figure 3B). They will serve as a reference during the rest of the sample processing steps. In our case, we dehydrated the tongues after fixation, embedded them in paraffin, and sectioned the blocks with 4 μm thickness, followed by hematoxylin & eosin, Masson trichrome, immunocytochemistry, or immunofluorescence staining (Figure 3C, D). Histological sample processing is not described in this protocol. We processed the fresh frozen samples for tissue section AFM (Figure 3E) as described previously [28,29].

## Validation of protocol

Figure 3 presents examples of approaches and modalities to extract qualitative and quantitative data from the tongue samples.

Taking pictures and weighing the tongue samples fresh post-dissection allows a relatively quick quantification of obvious differences between tumor sizes among multiple groups [30]. This requires a camera to acquire images and a precision scale to measure the tongues (Figure 3A). However, this approach is crude and demands a reproducible dissection procedure to extract meaningful data.

Post-fixation with formalin, stereomicroscopes can be used to make more precise observations with no extra experimental costs (Figure 3B) [1,31]. If the tumor cells express fluorescent markers, the fluorescence signal intensity from these images can be quantified. Here, we show one non-injected control and a tumor-injected tongue. Using intensity-based segmentation, we created a region of interest corresponding to the whole tissue and calculated mean signal intensities (for GFP-expressing tumor cells).

After formalin fixation, paraffin embedding, and sectioning, we performed hematoxylin & eosin, Masson’s trichrome, and immunohistochemistry (for E-cadherin) staining using the aforementioned standard protocols (Figure 3C) [32–34]. These staining techniques allow histopathological analyses of tumor growth, its microenvironmental composition, and tumor invasion, among others. Furthermore, we performed immunofluorescence labeling of the sectioned tissue with an anti-GFP antibody to image the GFP-tagged tumor cells at high resolution (Figure 3D). Of note, the tongue tissue is strongly autofluorescent due to the presence of muscle fibers and red blood cells.

Finally, fresh-frozen sections embedded in OCT after dissection were stained, and tissue/tumor stiffnesses were measured using AFM (Figure 3E) [35–37]. Tongue samples were cut into 30-μm thick cryosections at -20 °C, immediately transferred to a poly-L-lysine-coated glass-bottom dish, and immersed in PBS-containing cOmplete EDTA-free protease cocktail. The sections were then incubated with 40 μg/mL CNA35-GFP [38–40] to label collagen and with 5 μg/mL DAPI in protease inhibitors containing PBS supplemented with 0.2% Triton X-100 (for 1 h at room temperature). The AFM measurements were done on the same day. Young’s modulus for each force curve was calculated using JPK software (JPK DP version 4.2), assuming a Hertz model of impact.

**Figure 3.**
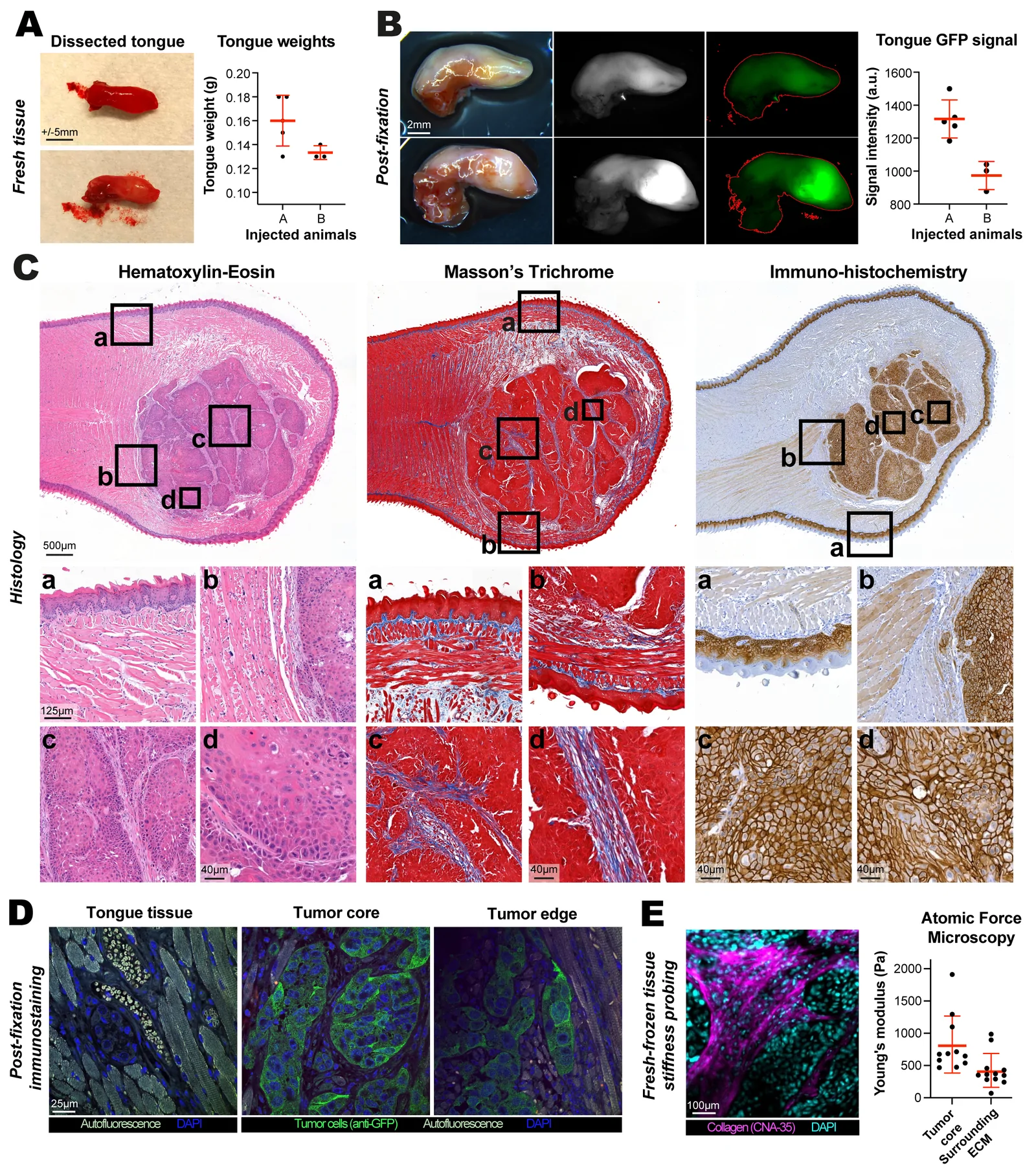
Anticipated result examples. (A) Freshly dissected tongue pictures (left) and a plot showing measured weights between two groups of injected animals (right). Only group A developed large tumors in this example. Data shown as single data points and mean ± standard deviation (SD). Scale bar is approximately 0.5 mm (phone camera). (B) Stereomicroscope picture of the tongue. Tongues were taken out of 70% ethanol (post-fixation solution) and placed in a plastic dish (dry) for rapid imaging before risking them to dry. (Left) Transmitted light acquisitions. (Middle) Fluorescent acquisitions of the same field of view. (Right) False color version of the middle image, with a red line depicting the result of the segmentation performed in ImageJ. Analysis using intensity-based thresholding methods allows the segmentation of the tongue area and the quantification of the signal inside the mask. Quantification shows the same groups A and B from panel A; this approach reflects the result of the experiment with more precision. Data shown as single data points and mean ± SD. Scale bar, 2 mm. C. Hematoxylin & eosin, Masson’s trichrome, and immunohistochemistry against E-cadherin staining of tongue slices. Low- and high-magnification pictures are shown. Scale bars, 500 μm for low magnification (top line); 125 μm and (if not indicated) 40 μm for high magnification. (D) Immunofluorescence labeling of a tongue section bearing a tumor with anti-GFP antibody. Scale bar, 25 μm. (E) Fresh-frozen section image from CNA-35-GFP and DAPI staining used for atomic force microscopy (AFM) measurement of the stiffness of the tumor core and surrounding extracellular matrix. Data shown as single data points and mean ± SD. Scale bar, 100 μm.

## General notes and troubleshooting

**General notes**

1. Mice to be included in the protocol:

a. We see no limitation in the strain and sex of the animals. These have to be adjusted to the specific objective of the experiment.

b. To accommodate the procedure, we recommend using healthy young adult mice over 18 mg. The animals will most probably undergo a temporary weight loss (Figure 1B); thus, they should be in optimal shape (no stress signs, no scratches, no pathogens) before the experiment.

c. Although it is up to the experimental objective, we do not recommend the inclusion of PBS- or Matrigel-injected mice as a control for this experiment. By the time of sacrifice, those substances have long been processed by the mouse tissue. We believe this means unnecessary extra pain for the animals, with no experimental benefit. Nevertheless, the comparison with healthy tissue has been very valuable for us, and we recommend including two non-injected animals at the sacrifice time.

d. Sample size must be decided by the experimenter. We recommend a minimum of six animals per experimental group, and a minimum of two independent repeats of the experiments. N = 12 biological repeats will allow you to perform quantitative analysis (t-test and ANOVA).

2. Cell concentration was empirically determined based on our experience with previously performed in vivo injection experiments. We obtained satisfying results and would recommend starting with 1 million cells per mL (1 × 10^6^ cells/mL), a relatively high concentration classically used in vivo. However, we also recommend testing several concentrations if needed. Injection cell suspension preparation depends on cell culture maintenance conditions (media used, trypsin concentration, time of trypsinization, etc.) and is not described in this protocol. Trypsinization, counting, and resuspension to the correct volume should be performed as usual with the chosen cell line. To achieve 30,000 cells/30 µL (injection volume), the calculation can be done with the equivalent of 1 million cells/mL. In case of a 30% dilution required when using Matrigel (or other solutions), prepare cells at a concentration of 1.43 million cells/mL such that 30,000 cells are injected after dilution.

3. Matrigel is optional, not required, as we never observed leakage of the cell suspension out of the tongue while retracting the needle after injection. Matrigel is a bioactive compound that can influence your experiments; hence, carefully assess your needs and consider alternative reagents. We used Matrigel in our experiments [9] to maximize the grafting success of our cells (previously tested in other in vivo experiments).

4. Expected timing for the completion of the protocol

a. Before the experiment (steps A1–2): 1 month for planning, mouse, and material delivery.

b. Intratongue injection day (steps B1–15): 1 full day for 20–30 animals; cell preparation, mice preparation, injections, and recovery post-anesthesia.

c. Monitoring after intratongue injection (steps C1–5): As long as the experiment lasts (anticipated time between 2 and 4 weeks). You need 30 min every day for daily monitoring and soft food changing. You need 2 h every week for tongue check (for 20–30 animals).

d. End of the experiment, sacrifice and dissection (steps D1–7): A half day of dissection for 20–30 animals. Some extra time the day after to transfer from formalin to 70% ethanol.

e. Anticipated results: From 1 day to 1 month, depending on the approaches.

**Troubleshooting**

**Table:** 

Step	Possible issue	Risk of occurrence for beginners (0 to 5/5)	Possible reason	Troubleshooting guide
B7	Matrigel sets during solution preparation	1	Matrigel will set at room temperature.	Never keep Matrigel out of ice. Keep the cell solution mixed with Matrigel also on ice.
B7	Matrigel sets in the injection needle	4	If intratongue injections are not done quickly, the Matrigel will solidify in the 30G syringe.	Keep the needle cold on the ice surface. Injection training with solutions that do not contain Matrigel is recommended to increase pace.
B11	Injection suspension leaks out of the tongue during injection	2	The needle transpierced the tongue, probably due to a wrong angle while injecting.	Carefully check Figure 1D. The needle entry should be nearly parallel to the tongue and only 2 mm in depth to stay in the mucosa.	
C1	Early animal death (day 1)	2	Total anesthesia can cause animal death.	There is no fix. Some animals do not recover from KX mix; hence, death is unpredictable. Plan extra animals.	
C1	Early animal death (days 1–3 post-injection)	2	Intratongue injection was performed too deeply in the mouth. The mouse cannot eat/drink properly.	Double-check Figure 1D. The injection point needs to be on one side and not too deeply in the mouth. Plan an extra animal for the first experiment.	
C1	Softened food stays in the cage for more than 24 h	1	The animals are *playing* with the food, and it is difficult to gather all the pieces of food from the previous day.	The use of a small bowl helps. Eliminating all past-day food is important to limit the risk of rotting. Changing the cage more often than usual is advisable.	
C1	Animal death (from day 3 post-injection to endpoint)	1	Unpredictable choking during eating.	Make sure the softened food is entirely soft. Closely monitor the animals. Any sluggish animal should be manipulated, and its mouth should be checked.	
C2	Stressed animals during the protocol, showing aggressive behavior against each other	4	The presence of a tumor early after injection and close to the endpoint is inducing animal stress.	There is no complete fix. Using PAI mix for extra days post-injection or near the end of the protocol can help the animals. Careful monitoring is necessary.	
C4	Difficulties in taking good pictures of the tongues	4	No free hands to take pictures.	Ask for help from a colleague or animal caretaker to take pictures while you manipulate the animals.	
D1	Difficulties in predicting the endpoint	3	If the endpoint is based on the health status of the animal, the tumor will likely grow to a size that impairs the uptake of food.	Monitor the animals very carefully toward the end of the experiment. Weigh the animals every day if needed. Be strict about sacrificing, as animals can die very quickly once they start losing weight.	
D4	Dissection difficulties	2	This dissection is not very common; cutting the lower jaw.	Use good scissors to confidently cut the jaw joint. Point the scissors from the opening of the mouth toward the foreleg of the animal while making the incision (Figure 2A).	
D4	Dissection difficulties	2	This dissection is not very common; cutting the tongue out.	It is easier to get the tongue sample in two steps, as described: first, take a larger tissue out of the animal and into the PBS. Second, clean and refine the sample.	
